# Supplementary material for: Adenosine leakage from perforin-burst extracellular vesicles inhibits perforin secretion by cytotoxic T-lymphocytes
Source: PLoS One. 2020 Apr 10;15(4):e0231430. doi: 10.1371/journal.pone.0231430 (PMC7147783; doi:10.1371/journal.pone.0231430)
Supplement: S1 Raw images — (PDF) [file pone.0231430.s001.pdf]

S1 Fig. C:

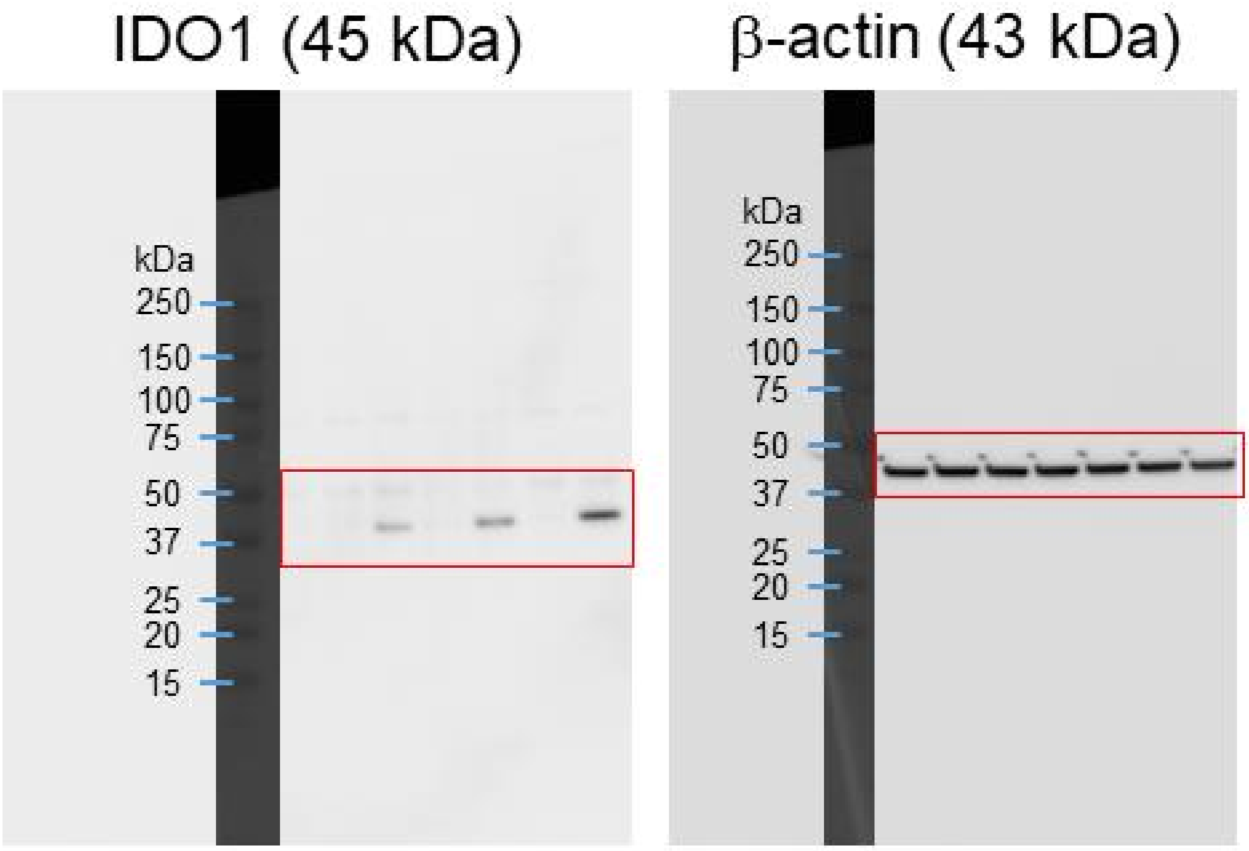

S1 Fig. E (Left):

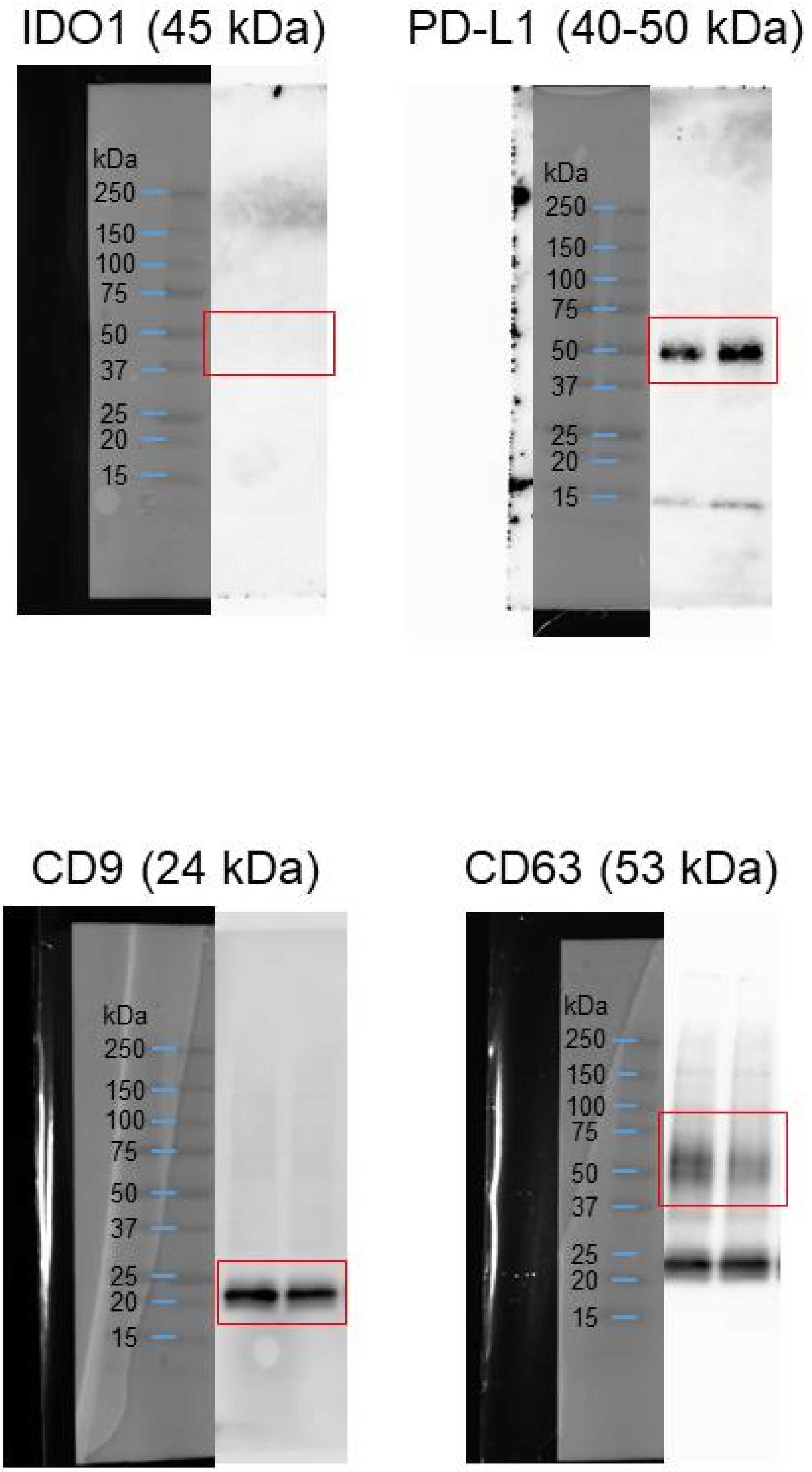

IDO1 (45 kDa)
PD-L1 (40-50 kDa)
CD9 (24 kDa)
CD63 (53 kDa)
kDa
250
150
100
75
50
37
25
20
15

S1 Fig. E (Right):

Alix (85 kDa)

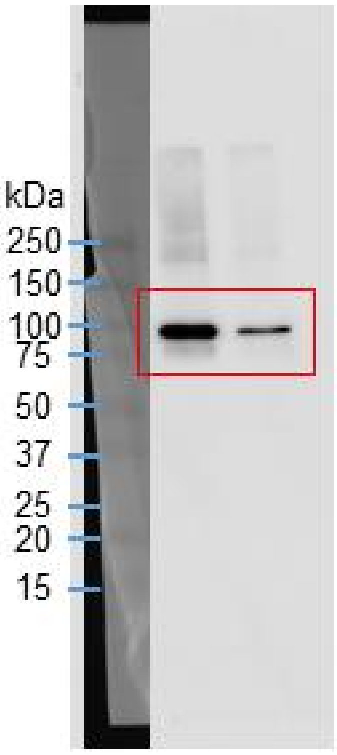

Flotillin-1 (48 kDa)

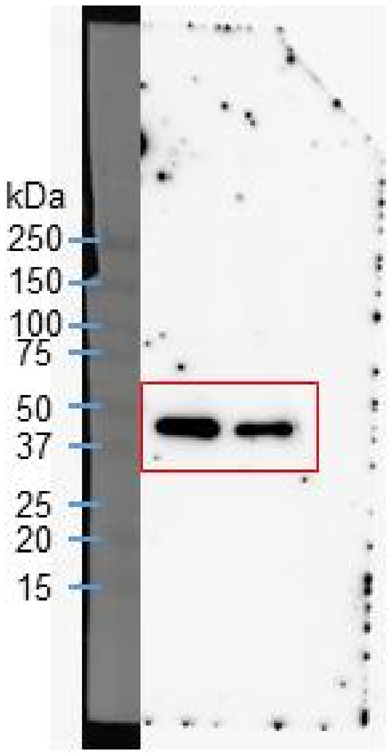

TSG101 (46 kDa)

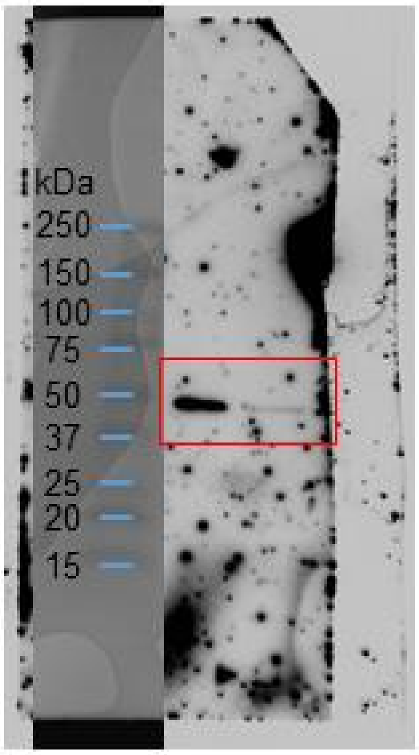

APOA1 (28 kDa)

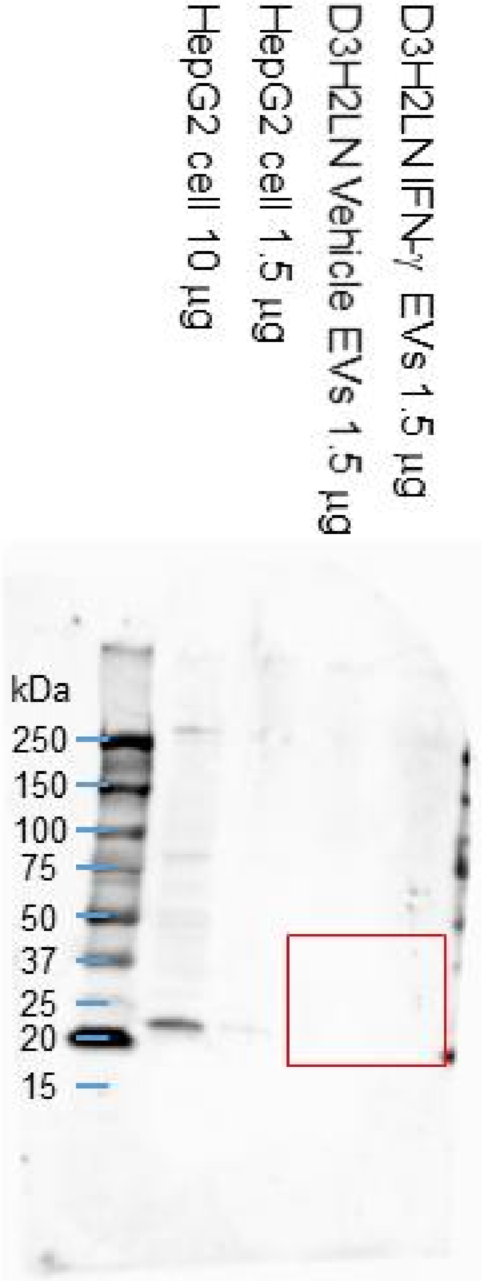

HepG2: Positive control
