# Supplementary material for: Adenosine leakage from perforin-burst extracellular vesicles inhibits perforin secretion by cytotoxic T-lymphocytes
Source: PLoS One. 2020 Apr 10;15(4):e0231430. doi: 10.1371/journal.pone.0231430 (PMC7147783; doi:10.1371/journal.pone.0231430)
Supplement: S1 Table — From the metabolome profiling results for each EV, the proportion of each metabolite in the total metabolites of each EV was calculated, and the number of metabolites accounts for each proportion range was indicated. (DOCX) [file pone.0231430.s002.docx]

**S1 Table. Number of EV metabolites detected in each rate range**

|  | Number of metabolites | |
| --- | --- | --- |
| % | Vehicle EVs | IFN-γ EVs |
| > 3.0 % | 10 | 7 |
| 2.9 to 2.0 % | 5 | 7 |
| 1.9 to 1.0 % | 7 | 9 |
| 0.9 to 0.0 % | 49 | 48 |
| total | 71 | 71 |
|  |  |  |

From the metabolome profiling results for each EV, the proportion of each metabolite in the total metabolites of each EV was calculated, and the number of metabolites accounts for each proportion range was indicated.
