# Supplementary figures and images for: Adenosine leakage from perforin-burst extracellular vesicles inhibits perforin secretion by cytotoxic T-lymphocytes
Source: PLoS One. 2020 Apr 10;15(4):e0231430. doi: 10.1371/journal.pone.0231430 (PMC7147783; doi:10.1371/journal.pone.0231430)

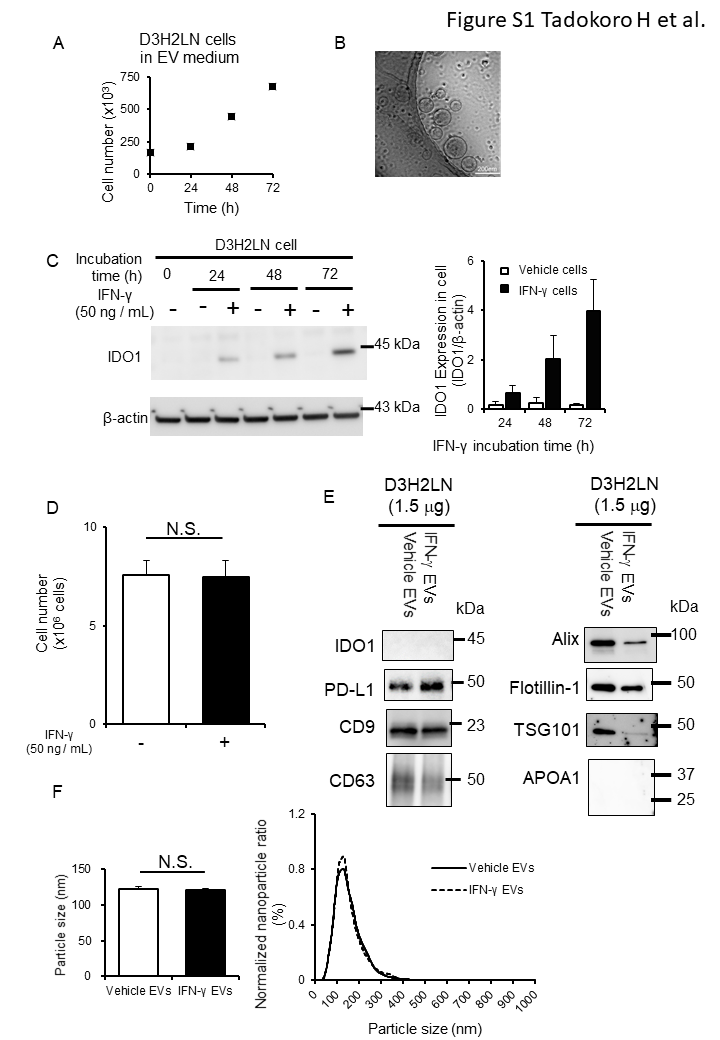

Supplement: S1 Fig — To establish an experimental model to identify immunosuppressive metabolites, we first confirmed the responses of D3H2LN cells to IFN-γ. (A) Growth of D3H2LN cells in EV medium. D3H2LN cells (1.0 × 10^5^ cells/6 well) were seeded and cultured overnight in maintenance medium. After the medium was changed to EV medium, cell growth was measured at 0, 24, 48, and 72 h. (B) The transmission electron microscope image shows D3H2LN EVs 48 h after the medium was changed to EV medium. (C) Expression of indoleamine 2,3- dioxygenase 1 (IDO1) protein by D3H2LN cells cultured with or without IFN-γ for 24, 48, and 72 h. Bands on immunoblots were quantified using ImageJ software (http://rsb.info.nih.gov/nih-image/). (D) Number of D3H2LN cells. D3H2LN cells (3 × 10^6^ cells/150 mm2) were seeded and cultured with or without IFN-γ. The number of cells was counted after 48 h. (E) Expression of indoleamine. 2,3-dioxygenase 1 (IDO1), programmed death-ligand 1 (PD-L1), CD9, CD63, Alix, Flotillin-1, TSG101, and apolipoprotein A1 (APOA1) in EVs from D3H2LN cells treated with or without IFN-γ. EV markers, CD9, CD63, Alix, Flotillin-1, and TSG101; EV negative protein marker, APOA1; immunosuppressive markers, IDO1 and PD-L1. (F) Average size of each EV, as assessed by NTA (left panel). As an example, size distribution of EVs derived from D3H2LN cells treated with or without IFN-γ for 48 h was assessed by NTA. All experiments were performed using three biological replicates. (TIF) [file pone.0231430.s003.TIF]

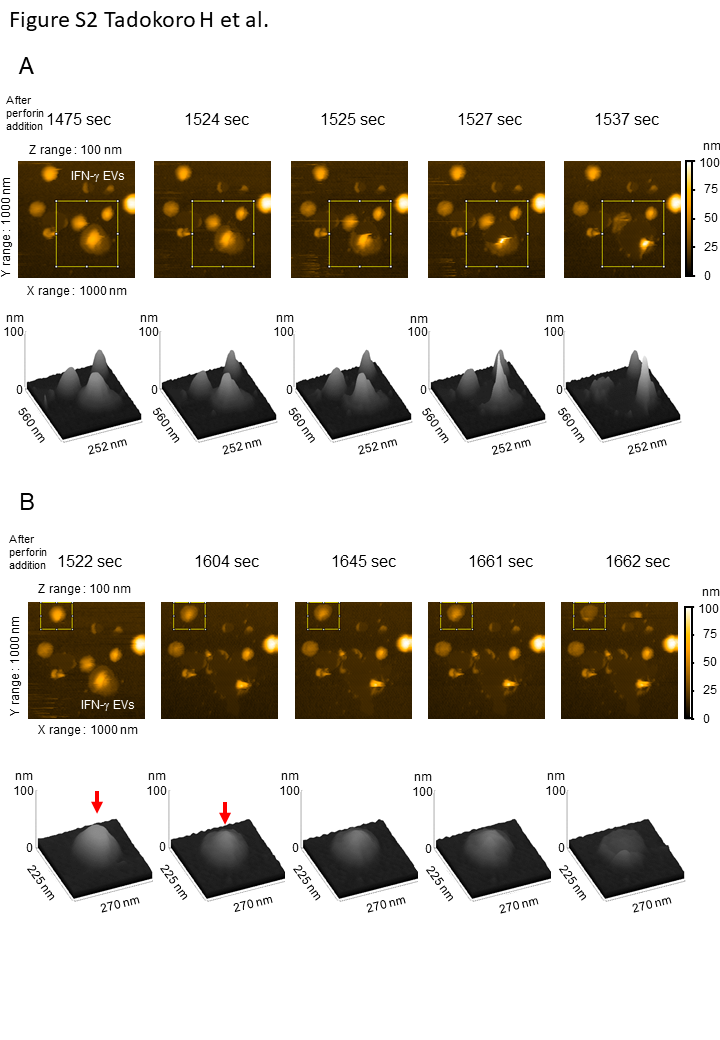

Supplement: S2 Fig — A solution of EVs from IFN-γ-treated D3H2LN cells (IFN-γ EVs) (14 ng in PBS) was incubated for 10 min on a mica surface in a humidified environment. Next, the mica was washed twice with PBS and imaged under an atomic force microscope (S4 Movie and S4 3D Movie: (A), S5 Movie and S5 3D Movie: (B)). USC-F1.2-k0.15 was used as a cantilever. B. Some of EVs from IFN-γ-treated D3H2LN cells (IFN-γ EVs) shrunk before bursting in response to perforin. The 3D images (processed using ImageJ software) derived from successive AFM images show a height reduction in IFN-γ EVs (red arrow). These images were obtained from the sample shown in Fig 2 but from a different area. (TIF) [file pone.0231430.s004.TIF]

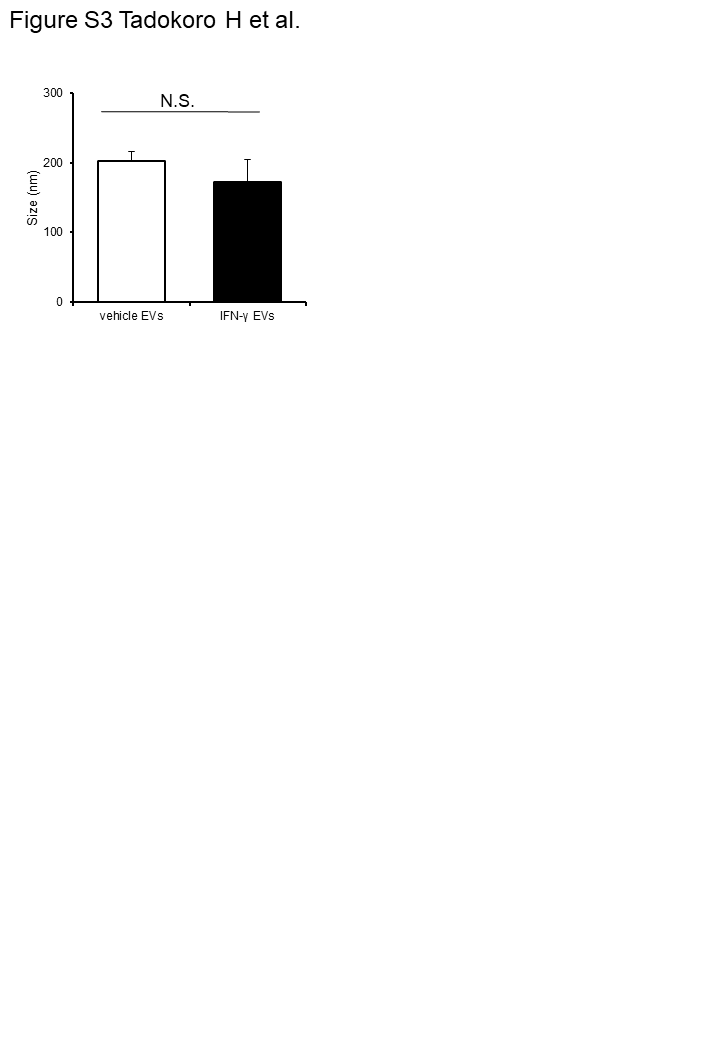

Supplement: S3 Fig — The diameters were calculated from cross-sectional image analysis of each AFM photo using ImageJ software. For this analysis, five vehicle EVs and eight IFN-γ EVs were analyzed. (TIF) [file pone.0231430.s005.TIF]

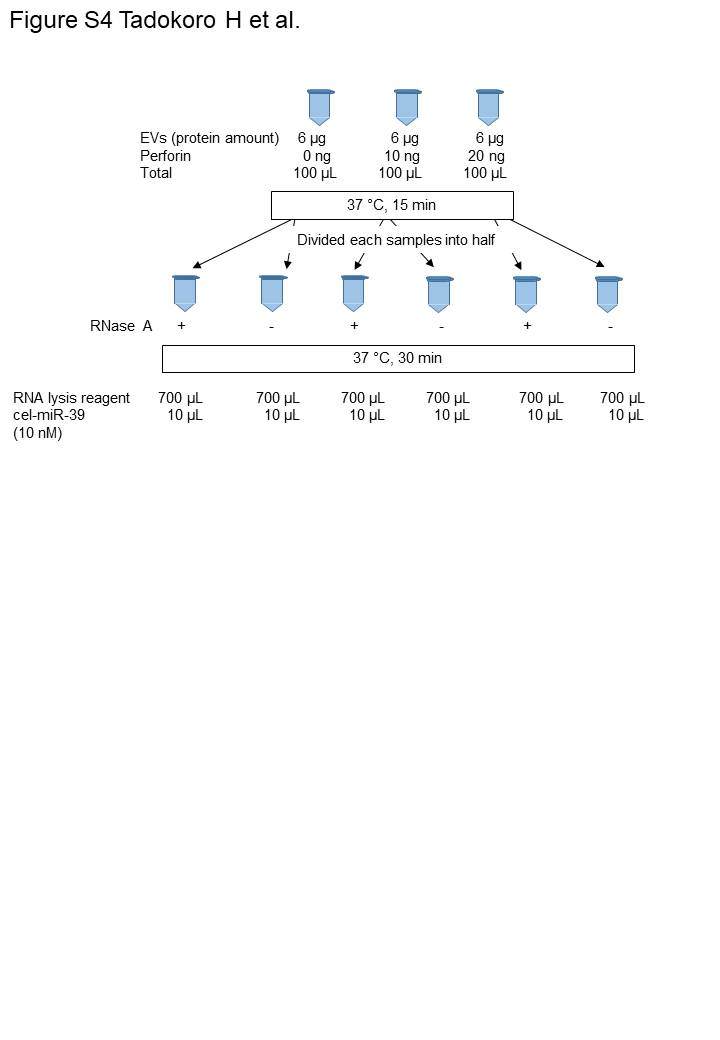

Supplement: S4 Fig — Formation of perforin pores in the membrane of EVs from vehicle-treated D3H2LN cells (vehicle EVs) and EVs from IFN-γ-treated D3H2LN cells (IFN-γ EVs) was measured indirectly using real-time PCR to detect miR-16. Both types of EV were treated with perforin (0, 100, or 200 ng/mL) in the presence or absence of RNase A. After addition of an RNA lysis reagent, samples were spiked with cel-miR-39. (TIF) [file pone.0231430.s006.TIF]

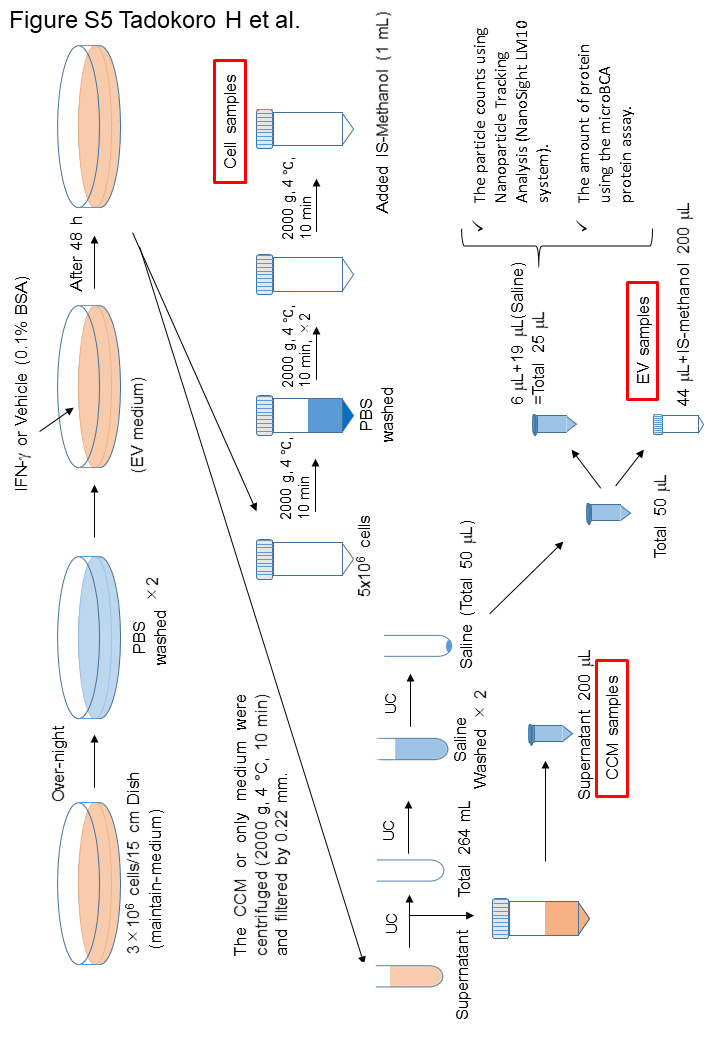

Supplement: S5 Fig — Cell samples, cell culture medium (CCM) samples, and EV samples for metabolomic profiling were collected according to this scheme. The EV medium was used as an indication of the background metabolic signature of the culture medium. UC, ultracentrifugation (110,000g for 70min at 4°C); IS, internal standard. (TIF) [file pone.0231430.s007.TIF]

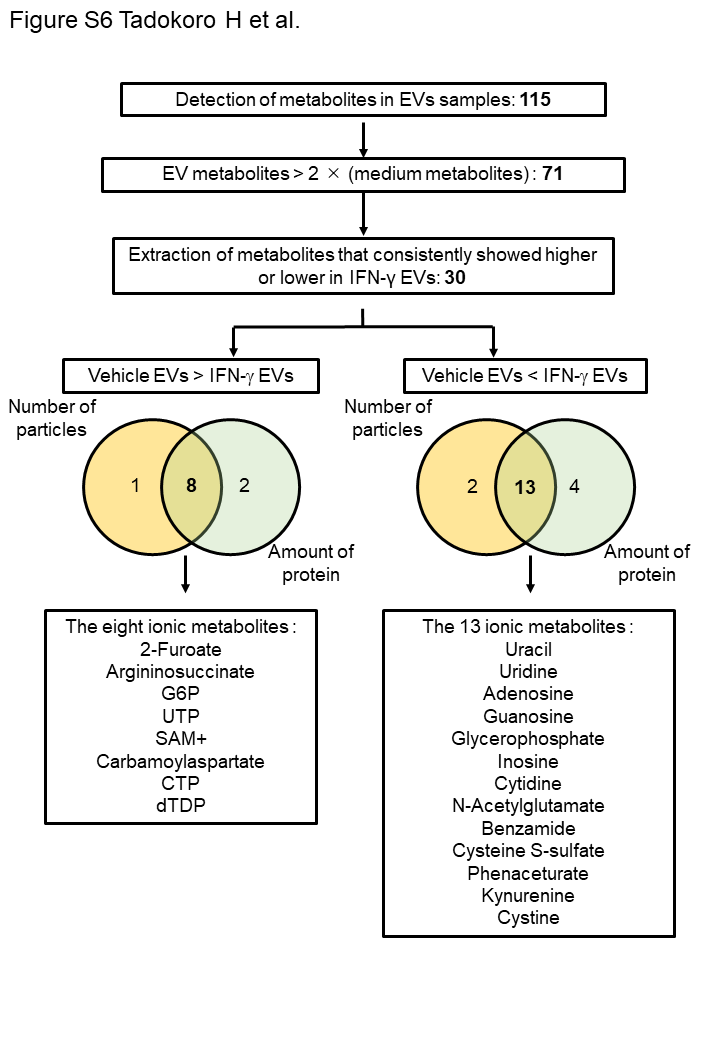

Supplement: S6 Fig — One-hundred-and-fifteen metabolites were detected in EV samples. Of these, 71 that were present in the EV samples at levels above twice the level detected in the blank were selected. Next, 30 metabolites present at consistently higher or lower levels in IFN-γ EVs were extracted. These metabolites were normalized in two ways: against particle number and against the amount of protein. Metabolites present in higher amounts in vehicle EVs than in IFN-γ EVs were detected by normalization against particles (n = 9) and by normalization against the amount of protein (n = 10). Of these, eight were detected after normalization against both particle number and amount of protein. These eight metabolites were not listed in the top ten metabolites detected according to absolute contribution rate values (Table 2). The total number of metabolites in IFN-γ EVs that exceeded those in vehicle EVs normalization against particles was 15, whereas the number after normalization against protein amount was 17; of these, 13 were detected by normalization against both particle number and protein amount. Of these 13 metabolites, five (uracil, uridine, adenosine, guanosine, and inosine) were listed in the top ten metabolites detected according to absolute contribution rate values (Table 2). All experiments were performed using three biological replicates. (TIF) [file pone.0231430.s008.TIF]

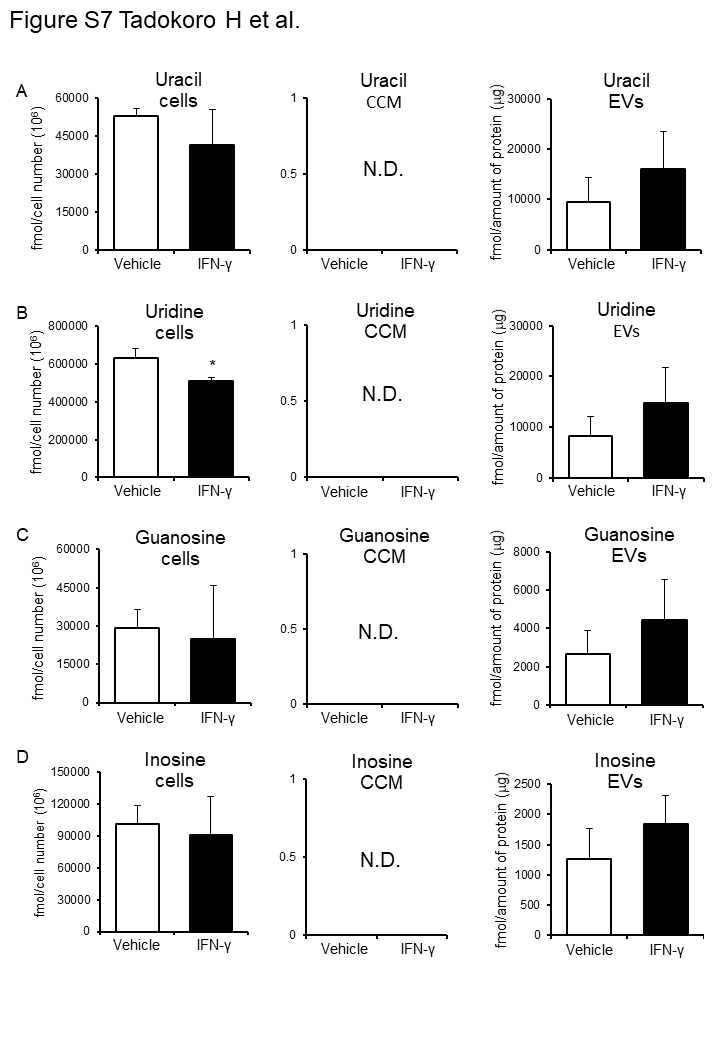

Supplement: S7 Fig — Cells and cell culture medium (CCM) samples were analyzed by CE-MS, and EV samples were analyzed by IC-MS and LC-MS. Cells and CCM samples were also analyzed using LC-MS to detect uracil; this is because uracil is not detected accurately by CE-MS. A. Amount of uracil in cells, CCM, and EVs. B. Amount of uridine in cells, CCM, and EVs. C. Amount of guanosine in cells, CCM, and EVs. D. Amount of inosine in cells, CCM, and EVs. Columns, average concentration (cell samples; fmol/cell number (106); EV samples; fmol/mg protein); bars, S.D. (n = 3). N.D., the metabolite concentration was below the detection limit of the assay. P < 0.05 (Student’s t-test). All experiments were performed using three biological replicates. (TIF) [file pone.0231430.s009.TIF]
