# Supplementary material for: Adenosine leakage from perforin-burst extracellular vesicles inhibits perforin secretion by cytotoxic T-lymphocytes
Source: PLoS One. 2020 Apr 10;15(4):e0231430. doi: 10.1371/journal.pone.0231430 (PMC7147783; doi:10.1371/journal.pone.0231430)
Supplement: Attachment — Submitted filename: Response_to_Reviewers_Tadokoro_PONE-D-19-35246.docx [file pone.0231430.s020.docx]

**Point-by-point responses to each of the reviewers’ comments**

We wish to thank the reviewers for the insightful comments, which have helped us improve the paper. As indicated in the responses that follow, we have taken all of these comments and suggestions into account when revising the paper, including the supplementary information. All changes in the "Revised Manuscript with Track Changes" are highlighted in red.

**Responses to Journal Requirements**

2. Please provide additional details regarding participant consent and IRB approval for sampling of human PBMCs. In the ethics statement in the Methods and online submission information, please ensure that you have specified (1) whether consent was suitably informed and (2) what type you obtained (for instance, written or verbal). If your study included minors under age 18, state whether you obtained consent from parents or guardians. If the need for consent was waived by the ethics committee, please include this information.

**Response:** The human PBMCs used in the study were purchased from STEMCELL Technologies, Vancouver, Canada. In the "Commitment to Quality" section of the company website, STEMCELL Technologies states that “cells are collected using Informed Consent Forms (ICFs) approved by either the Food and Drug Administration (FDA) or an Institutional Review Board (IRB), ensuring ethical sourcing while protecting donor anonymity.”

3. In your Methods section, please provide additional details regarding the cell lines

used in your study and ensure you have described the source.

**Response:** In accordance with the editor's comment, we have added information about the company supplying the MDA-MB-231-luc-D3H2LN cell line (see the “Cells and culture conditions” subsection of the "Materials and methods" section; p.5, lines 85–86) as follows: "The breast cancer cell line MDA-MB-231-luc-D3H2LN (D3H2LN) (Xenogen Corporation, California, USA)".

4. We note that you have included the phrase “data not shown” in your manuscript.

Unfortunately, this does not meet our data sharing requirements. PLOS does not permit references to inaccessible data. We require that authors provide all relevant data within the paper, Supporting Information files, or in an acceptable, public repository. Please add a citation to support this phrase or upload the data that corresponds with these findings to a stable repository (such as Figshare or Dryad) and provide and URLs, DOIs, or accession numbers that may be used to access these data. Or, if the data are not a core part of the research being presented in your study, we ask that you remove the phrase that refers to these data.

**Response:** We have removed this sentence from the text.

**Responses to the comments of Reviewer #1**

Reviewer #1: The authors have attempted to elucidate the mechanisms employed by breast cancer cells derived EVs to overcome immune attack by CTLs. Here they show that perforin opens up EV membranes and results in adenosine release which can suppress CTL mediated perforin secretion. They also show the alleviation of this inhibition by the addition of ADA.

I believe that the authors need to address a few major points for the manuscript to be suitable for publication

1) Please edit your manuscript for sentence structure and some minor grammar. The text needs to be uniformly in past tense unless speculating or discussing your data.

**Response:** Thank you. The paper has been edited by an experienced scientific editor, who has improved the grammar and stylistic expression of the paper.

2) Please include an explanation of

2)-a, why breast cancer cells were chosen for this study?

2)-b, Secondly, why did the authors limit their study to just one cell line. They could have easily included multiple breast cancer cell lines or alternately, looked at this phenomenon across tissue types

**Response:** We wish to thank the reviewer for this comment.

2)-a: There are two reasons why we used cell line MDA-MB-231-luc-D3H2LN. One is that this cell line secretes a large number of EVs. Another is that this cell line shows changes in metabolism (e.g., catabolism of tryptophan by IDO1) upon exposure to IFN-γ. As we mentioned in the “Abstract” and “Introduction”, we hypothesized that EV metabolites have an immunosuppressive function. Therefore, we preferred to use this cell line, in which changes in cellular metabolism can be controlled.

2)-b: We also think that it is a limitation of the study; however, we believe that perforin-mediated EV destruction is quite interesting and would like to be the first to publish this observation. As the reviewer mentions, it is better to use several cell lines to confirm this phenomenon; therefore, we have mentioned this as a limitation in the “Discussion” section (p.29, lines 499–500) as follows: “The third limitation is that the results are not generalizable because they are based on only one cell line.” Thank you.

3)-a, DO the authors exclude all serum (FBS) from their EV medium?

3)-b, Does that impact the cellular health as opposed to growing them in exosome depleted FBS?

**Response:** We appreciate the reviewer's comment on this point.

3)-a: Yes, we used advanced RPMI-1640 medium (Thermo Fisher Scientific) supplemented with 2 mM L-glutamine (Thermo Fisher Scientific) and Antibiotic-Antimycotic (1×). Previously, we optimized EV collection conditions using this medium [1–3]; MDA-MB-231-luc-D3H2LN grows under these conditions.

3)-b: As we stated in 3)-a, we used advanced RPMI-1640 medium (Thermo Fisher Scientific) supplemented with 2 mM L-glutamine (Thermo Fisher Scientific) and Antibiotic-Antimycotic (1×) for EV collection. We have not compared it with exosome-depleted FBS. Thus, we do not have a specific answer to this question.

As shown below, MDA-MB-231-luc-D3H2LN cells grow in EV medium.

D3H2LN cells in EV medium

Cell number (x103)

| Time (h) | D3H2LN |
| --- | --- |
| 0 | 168.333 |
| 24 | 211 |
| 48 | 444.5 |
| 72 | 674.352 |

In accordance with the reviewer's comment, we have included this figure as S Fig 1A. The legend states: “(A) Growth of D3H2LN cells in EV medium. D3H2LN cells (1.0 × 105 cells/6 well) were seeded and cultured overnight in maintenance medium. After the medium was changed to EV medium, cell growth was measured at 0, 24, 48, and 72 h.” (p.40, lines 697–699).

4) Please edit the sub-section "sample collection and metabolite extraction:" within the methods section. It is unclear what the authors have processed in this section. Perhaps a schematic might be useful?

**Response:** Thank you. We have included a Figure and the following legend: “**Scheme showing sample collection and metabolite extraction.** Cell samples, cell culture medium (CCM) samples, and EV samples for metabolomic profiling were collected according to this scheme. The EV medium was used as an indication of the background metabolic signature of the culture medium. UC, ultracentrifugation (110,000 g for 70 min at 4°C); IS, internal standard.” (p.42, lines 736–740).

We also changed the description to “Vehicle EVs and IFN-γ EVs (collected according to the scheme shown in Fig S5), were subjected to metabolome analysis using LC-MS.” This is stated in the “Metabolomic profiling of IFN-γ EVs” subsection of the "Results" (p.18, lines 320–321).

5) The movies included are very interesting, One suggestion would be to speed them up to 10X so that the viewers are not waiting for a long time for EV lysis.

**Response:** We wish to thank the reviewer for this suggestion. We have edited the movies and speeded them up by 10 times. This is now mentioned in all relevant legends: “All images were taken at 1 frame per second (fps) and were edited at 10× speed (10 fps) using ImageJ.”

All legends for 3D movies now include the following sentence: "All 8-bit grayscale photos were taken at 10 fps."

6) Is it significant that the IFN EVs take longer to burst than the vehicle EVs? ( 7mins compared to 30 secs and 3 mins?

**Response:** The time difference with respect to the effects of perforin on IFN-γ EVs and vehicle EVs is interesting. However, both vehicle EVs and IFN-γ EVs burst at around 7 mins after addition of perforin (Fig. 1), whereas other IFN-γ EVs burst almost 25 mins after addition of perforin (S2 Fig).

High-speed atomic force microscopy (HS-AFM) is not well-suited for comparing the timing at which each type of EV bursts. To acquire a burst image using HS-AFM, one must first identify a suitable EV on the stage and then continue observing it until it bursts. We cannot observe other EVs while waiting for one to burst. Therefore, the burst time of the last observed EV will affect the burst time of subsequent EVs.

We mentioned this in the "Discussion" section (pp.26-27, lines 447–449); however, we have now revised the text as follows: “The kinetics of EV contraction and perforin destruction must be investigated in future, by observing a wider area at AFM-level super-resolution.” (p.27, lines 449–451). We appreciate the reviewer's comment.

7) Consider combining both the panels in figures 2A and 2B into one. A stacked bar plot can express the data equally well and allow for comparisons between conditions

**Response:** We apologize for the confusion. We believe that it is not appropriate to combine Figure 2A and 2B because vehicle EVs and IFN-γ EVs do not contain the same amounts of miR-16. Also, we revised the text in the “Discussion” section as follows: “Since miR-16 is used as a housekeeping miRNA in cells, it was used as a marker for membrane disruption due to its high abundance in EVs.” (p.29, lines 490–491).

In the section "Metabolomic profiling of EVs"

8) Table 1 shows metabolites detected at >3%. However. The totals per group of EV is less than 70%. Does this mean that the remaining 30% were comprised of metabolites at very low concentrations? Please comment on this for both vehicle and IFN Evs

**Response:** Yes, both EVs contained many types of metabolite at very low concentrations. We have included a table showing the number of EV metabolites that account for each percentage range within the total amount of metabolites for each EV (see the Table below). In accordance with the reviewer's comment, we have revised the text as follows: “The concentrations of most metabolites derived from vehicle EVs and IFN-γ EVs were very low, accounting for only 0–0.9% of total EV metabolites (S1 Table).” (see “Metabolomic profiling of IFN-γ EVs” in the "Results" section; p.19, lines 327–329). We also added the table below as S1 Table (p.40, lines 689–692). We wish to thank the editor for this comment.

**S1 Table. Number of EV metabolites detected in each rate range**

|  | Number of metabolites | |
| --- | --- | --- |
| % | Vehicle EVs | IFN- γ EVs |
| > 3.0 % | 10 | 7 |
| 2.9 to 2.0 % | 5 | 7 |
| 1.9 to 1.0 % | 7 | 9 |
| 0.9 to 0.0 % | 49 | 48 |
| total | 71 | 71 |
|  |  |  |

From the metabolome profiling results for each EV, the proportion of metabolites in total was calculated, and the number of metabolites account for each proportion range was indicated.

9) Per Table 2, all metabolites are increased or enriched after IFN addition (since all values are positive). Table 1 shows that at least for UDP-N_acetylglucosamine and ADP that it is not the case. How can we reconcile the contribution rates with table 1? Also shouldn’t some metabolites be decreased in IFN EVs when compared to vehicle EVs after being normalized to total metabolites (per table 2 caption)

**Response:** We thank the reviewer for this comment. We mentioned that "Metabolites highlighted in bold had a positive contribution rate" in the footnote to Table 2. A positive contribution means that the metabolite was more contained in IFN-γ EVs than vehicle EVs. As the reviewer indicates, vehicle EVs contained more ADP than IFN-γ EVs. We have revised the footnote to Table 2 as follows: “Metabolites highlighted in bold were more contained in IFN-γ EVs than in vehicle EVs.” (p.22, lines 380–381).

IFN-γ EVs contained more **UDP-N-acetylglucosamine** than vehicle EVs; therefore, UDP-N-acetylglucosamine is highlighted in bold. The average amount of UDP-N-acetylglucosamine in IFN-γ EVs was 375.8 nM, whereas that in vehicle EVs was 349.7 nM. The percentages in Table 1 indicated the rate of metabolite in the total metabolites of each EV. The total amount of each metabolite is different in each EV; therefore, the percentage of each metabolite cannot be compared between vehicle EVs and IFN-γ EVs. We added the following description in the footnote to Table 1: “The percentage of each metabolite in both types of EV cannot be compared directly because the average amount of total metabolites in IFN-γ EVs was 2.4 µM, whereas that in vehicle EVs was 1.9 µM.” (p.21, lines 364–366).

10) Please edit line 319 where you reference Fig 3D (Fig 3 has not sub figures)

**Response:** We thank the reviewer for this comment. This error has been corrected.

11) In lines 321 and 322: why are 3 values being reported? Please report average values and standard deviations

**Response:** We apologize for the confusion. We reported three values to indicate the possibility that the presence of kynurenine in EV samples was due to contamination by the cell culture medium.

In accordance with the reviewer's comment, we changed the text as follows: “The concentration of kynurenine in IFN-γ EVs was 1.71, 18.25, and 8.90 nM (9.62 ± 6.77 nM) (Table 3). However, the concentration of kynurenine in IFN-γ CCM was 1.60, 1.57, and 1.84 µM (1.67 ± 0.12 µM).” (see "Discussion" section; p.20, lines 347–349). We also included Table 3 (p.23, lines 386–391). We appreciate the reviewer's comment on this point.

**Table 3. Three data sets: IFN-γ EV kynurenine content, IFN-γ CCM kynurenine content, and IFN-γ EV particle count**

|  | **1st** | **2nd** | **3rd** |
| --- | --- | --- | --- |
| **The kynurenine amount of IFN-γ CCM (nM)** | **1600** | **1570** | **1840** |
| **The kynurenine amount of IFN-γ EVs (nM)** | **1.71** | **18.25** | **8.9** |
| **The particle number of IFN-γ EVs (E^10^)** | **1.39** | **1.53** | **1.84** |

Metabolome analysis was performed using three biological replicates. Each EV was isolated from the same sample taken from each cell culture medium (i.e., 1st EVs were isolated from the 1st cell culture medium (CCM)).

12) The authors have not characterized their EVs per MISEV 2018 guidelines. Please add the necessary characterizations

**Response:** We thank the reviewer for this comment. In accordance with the MISEV 2018 guidelines, we have added the transmission electron microscopy images of EV, EV markers (Alix, Flotillin-1, and TSG101), and EV the negative protein marker APOA1 to S1 Fig.

We mentioned that “The EVs were characterized according to the MISEV 2018 guidelines [12]." in "Preparation of the EV fraction" section (p7, lines 116–117).

We added the following to the legend of S1 Fig. A: “(B) The transmission electron microscope image shows D3H2LN EVs 48 h after the medium was changed to EV medium.” (p.41, lines 700–701).

We also added the following description in the "Materials and methods" section: “ Isolated EVs were visualized by Terabase Inc. (Aichi, Japan) using a phase-contrast transmission electron microscope, which generates high-contrast images of the nanostructures comprising soft materials (including biological samples such as liposomes, viruses, bacteria, and cells) without the need for staining processes that may damage samples. The natural structure of a sample distributed in solution can be observed by preparing the sample using a rapid vitreous ice-embedding method, followed by cryo-phase-contrast transmission electron microscopy.” in “Phase-contrast transmission electron microscopy” section (p7, lines 118–125).

We also revised the legend to S1 Fig, E as follows: “(E) Expression of indoleamine. 2,3-dioxygenase 1 (IDO1), programmed death-ligand 1 (PD-L1), CD9, CD63, Alix, Flotillin-1, TSG101, and apolipoprotein A1 (APOA1) in EVs from D3H2LN cells treated with or without IFN-γ. EV markers, CD9, CD63, Alix, Flotillin-1, and TSG101; EV negative protein marker, APOA1; immunosuppressive markers, IDO1 and PD-L1. ” (p41, lines 706–710).

Some EV markers (CD63, Alix, Flotillin-1, and TSG101) were decreased in IFN-γ EVs compared with vehicle EVs. Villarroya-Beltri *et al.* reported that the interferon-stimulated gene 15 (ISG15) expression by IFN-I exposure inhibited the secretion of the classical exosome markers CD63, TSG101 and CD81 in the purified EVs [4]. In addition, IFN-γ exposure enhanced free ISG15 and ISGylation levels in MDA-MB-231 cells [5]. These mechanisms may have caused a reduction in these EV markers. However, in our study, we don't mention the effect of IFN-γ exposure on EV secretion.

13) The authors refer to the measurement of pore sizes formed by perforin. Is it possible to measure the pore size induced in the EVs membranes by perforin? How do they compare to the sizes reported in literature (for cells)

**Response:** We apologize for the confusion. The "pore size" mentioned in the “Discussion” (p.26, line 440) refers to the pore formed by polymerized perforin, not the size of the lyzed membrane hole. AFM could not reveal perforin polymerization due to technical problems. To avoid reader confusion, we have revised the text as follows: “We speculate that these differences are related to the pore size of polymerized perforin.” (p.26, lines 440–441).

In addition, we measured the pore size (size of the lyzed membrane hole) in the EV membranes using ImageJ, as described previously [6]. However, the reliability of this result is doubtful. The pore size of the cells examined in [6] did not change because the cells were fixed with 4% paraformaldehyde prior to imaging with AFM. We did not fix the EVs; therefore, the pore size in the EV membranes changed over time. Therefore, the pore size measured was dependent on the timing of the analysis.

We tried to measure pore sizes at the time of EV burst in each movie. The average pore size in vehicle EVs (two in movie S2 and additional photo in this letter) was 64.8 nm, whereas that in IFN-γ EVs (movies S3, S4, and S5) was 101.7 nm. There were no significant difference in the pore sizes of vehicle EVs and IFN-γ EVs (*P* = 0.09).

- Vehicle EVs
- S2 Movie at 473 sec after perforin addition

- S2 Movie at 620 sec

- Additional Vehicle EV measured at 890 sec after perforin addition

- IFN-γ EVs
- S3 Movie at 442 sec after perforin addition

- S4 Movie at 1525 sec after perforin addition

- S5 Movie at 1663 sec after perforin addition

- The average pore size in each type of EV

14) The authors state that IFNG stimulation does dramatically change the metabolites in EVs. This could be true, but the authors need to allow for the fact that their stimulation concentration, times and cell type not be optimal for the effects to be observed

**Response:** We think that the reviewer may have misunderstood. In the "Discussion", we stated that "IFN-γ treatment **did not** dramatically change the metabolites in EVs as much as cell metabolism." As noted by the reviewer, factors such as IFN-γ concentration, time, and cell type must be considered when deciding whether IFN-γ stimulation affects EV metabolites. However, in this paper we prefer to conclude that EV metabolites are not affected by intracellular metabolic alteration due to IFN-γ exposure.

We have revised the manuscript text as follows: “However, we found that IFN-γ treatment did not affect the metabolic profile in EV metabolites as much as cellular metabolites.” (see “Discussion" section; p.27, lines 459–460).

We have also made the following revision: “IFN-γ treatment promoted intracellular tryptophan catabolism and glycolysis; however, these differences were not apparent in EV metabolites between vehicle and IFN-γ treatment. In addition, the difference between metabolites in vehicle EVs and those in IFN-γ EVs were not observed. ” (see “Metabolomic profiling of IFN-γ EVs" in the “Results” section; p.20, lines 352–356).

15) The authors have shown an interesting response of CTL's to tumor derived EV's but as they admit, this needs to be validated in a mouse model to ensure that it is a biologically relevant mechanism. Additionally, as they point out, only 15% of the perforin secretion is reduced. Is the remaining 85% perforin secretion sufficient to cause lysis?

**Response:** Validation in an animal model is important; we think that establishing a new model is necessary. We think that the remaining 85% perforin secretion is sufficient for cell lysis. Although we do not have direct evidence, a reduction in the amount of perforin by 15% will have some effect on suppressing tumor apoptosis.

We have discussed the effect of a 15% reduction in perforin secretion in the context of other studies [7,8].

Shi et al. examined apoptosis of YAC-1 cells, a natural killer cell-sensitive mouse lymphoma cell line, in response to perforin (0–60 ng/mL) [7]. They showed that 20 ng/mL perforin induced apoptosis in 10% of YAC-1 cells, suggesting that a reduction in perforin levels of approximately 20 ng/mL may be effective on suppressing tumor apoptosis by perforin. In our study, EV adenosine suppressed perforin secretion by almost 15% (Fig 5A). The average perforin concentration in CTLs is 105.94 ng/mL; thus a 15% reduction amounts to almost 16 ng/mL perforin.

In addition, Chen et al. reported that tumor-derived EVs suppress perforin secretion, and that these EVs protect tumors from immune cells [8]. They examined the effect of RBE cell (a human cholangiocarcinoma cell line)-derived exosomes (TEX, tumor-derived exosomes) on perforin secretion by cytokine-induced killer cells (CIK) and on subsequent killing rates. TEX reduced perforin and TNF-α secretion by 30%, resulting in a 30% reduction in the killing rate by CIK cells. Thus, tumor-derived EVs can suppress both perforin secretion and CIK killing rates.

Thus, it appears that a reduction in the perforin concentration by almost 16 ng/mL may suppress apoptosis by almost 10%.

We appreciate the reviewer's comment on this point.

16) I assumed that the source of adenosine in the EVs is from the cells, but from the discussion it seems like cellular adenosine is short lived and rapidly converted to inosine. How is adenosine incorporated into EVs then?

**Response:** As we mentioned in the “Discussion”, the half-life of **extracellular** adenosine is very short (UNLIKE adenosine in EVs); this is because **extracellular** adenosine is taken up by cells, and it is degraded to inosine by adenosine deaminase. On the other hand, there is a certain amount of adenosine within cells because they synthesize it via that hydrolysis of AMP and methionine metabolism [9]. In addition, a review paper by Borowiec et al. shows that the reaction of phosphorylation (a reversible reaction) predominates when adenosine occurs at a low physiological concentration whereas adenosine deaminase is activated at higher concentrations of the substrate [10]. In cells, lysosomes contains high amounts of adenosine [11].

Our cell metabolic profiling results show that D3H2LN cells contain adenosine (Fig. 4A). The mechanism by which adenosine is incorporated into EVs remains unclear. However, one possibility is that EVs package adenosine because the cytosol contains adenosine.

We have added the following to the "Discussion" section (p.28, line 468). “The EV may package adenosine because the cytosol contains adenosine.”.

17) The authors should consider using multiple miRNA instead of just miR 16 for figure 2 in order to overcome the limitations of relying on a single miRNA to estimate EV integrity.

**Response:** We wish to thank the reviewer for this comment. In addition to miR-16, we used miR-210, which is abundant in EVs. The results for miR-210 showed the same trend as those for miR-16. In results of RNase A treatment samples, compared without perforin treatment EVs, perforin (200 ng/mL) led to a significant reduction in the amount of miR-210 in both IFN-γ EVs and vehicle EVs (Fig A, right panel and Fig B, right panel).

As we mentioned in the "Discussion”, the Ct value of miRNA effects the results. While the Ct value for miR-16 was between 29 and 35 (the average was 32.6), the Ct value for miR-210 was between 32 and 40 (the average was 34.9). Therefore, miR-210 is less accurate than miR-16; miR-16 is the best miRNA for this experiment because it is the most abundant in EVs.

**Figure. Measurement of the EV destruction by perforin using miR-210.** (A) EVs from vehicle-treated D3H2LN cells (vehicle EVs). (B) EVs from IFN-γ-treated D3H2LN cells (IFN-γ EVs). Both types of EV were treated with or without perforin, and miRNAs were degraded by RNase A (right panels). MiRNA degradation was measured by real-time PCR; cel-miR-39 was used as a spike-in control. *P* < 0.05 (Tukey–Kramer method). All experiments were performed using with three biological replicates, and each sample was compared with individual controls; thus the control has no standard deviation.

**Responses to the comments of Reviewer #2**

Reviewer #2: The manuscript “Self-sacrifice of cancer cell-derived extracellular vesicles leakage of adenosine disrupts perforin-mediated burst of extracellular vesicles by cytotoxic T lymphocytes” by Tadokoro et al., is a fluent and interesting read. As the authors address the young field of extracellular vesicles, the minuscule lipid membrane bound carriers of molecules, they are challenged by the uncertainties related to the success of purification and close-to-the detection limit data. However, the challenges are generally very well discussed, and therefore I feel that tackling some remaining issues would help the authors to provide a more comprehensive Fpublication. Here are my specific comments:

1) Title is too long and difficult to understand. In addition, the use of “self-sacrifice” sounds odd, when it describes the activity of EVs. Thus remove self-sacrifice and shorten the title.

**Response:** We wish to thank the reviewer for this comment. We have changed the title to “Adenosine leakage from perforin-burst extracellular vesicles inhibits perforin secretion by cytotoxic T-lymphocytes”.

2)-a, The data shows that perforin makes holes to some cancer-derived EVs (+/-IFNy treatment), which then leak adenosine. To provide more convincing data and knowledge of the extent of this phenomenon, the amount (e.g. the %) of bursting/shrinking EVs should be quantified.

2)-b, In addition, as a control, it would be good to show, whether perforin can perforate any EVs (fibroblast EVs etc +/-IFNy treatment) and whether the control EVs leak adenosine/reduce perforin secretion by CTLs as well.

**Response:** We appreciate the reviewer's comment on this point.

2)-a: We agree that quantification of bursting/shrinking EVs provides more convincing data. We measured the number of bursting EVs using real-time PCR (Fig 2). When we added perforin at 200 ng/mL, PCR did not detect almost 50% of the miR-16, suggesting that approximately 50% of EVs were damaged by perforin.

2)-b: Thank you very much for the suggestion. We agree that it is important to ascertain whether perforin can perforate any EVs. Theoretically, when CTLs recognize non-self-peptide/MHC class I complexes, they are activated and secrete perforin. Due to the non-self-peptide/MHC class I complex, perforin secreted from CTLs and NK cells may cause only cancer cell-derived EVs to rupture, whereas CTLs and NK cells may not secret perforin to fibroblast EVs in the body. In this study, we added exogenous perforin; therefore, the perforin was able to perforate any EV that it encountered. To investigate this further we require other models such as animal models. We have revised the manuscript text as follows: “Using the ovalbumin mouse model may help us to study whether perforin can perforate any EVs.” (see "Discussion" section; p.30, lines 511–512).

Whether control EVs leak adenosine/reduce perforin secretion by CTLs is another point. We think that this depends on how much adenosine is packaged in the EVs.

3)-a, Related to the previous, I found the miR assay results shown in Fig. 2A somewhat difficult to understand.

3)-b, Even if no RNAse is added, I would have expected that some natural RNAses remain in the EV preparations, that would degrade the miR-16 released from EVs when perforin is added. Instead, the quantification shows no degradation after addition of perforin.

3)-c, To support the AFM data about EV disruption, perhaps the best option would be to add some other data, for example to perform super-resolution imaging of EVs labeled with a soluble dye (+/- perforin).

**Response:**

3)-a: We apologize for the confusion. The graphs (left panels in A and B of Figure 2) are not significantly different. We added the abbreviation ‘N.S.’ to these graphs. The samples (100 and 200 ng/mL perforin) in Figure 2A (left panel) tend to be richer in miR-16 compared to 0 ng / mL perforin, but the result was not statistically significant.

3)-b: As the reviewer mentions, we cannot exclude the possibility of natural RNase contamination during EVs preparation. However, based on our data, the effects were negligible. Contamination by a small amount of RNase may degrade miR-16 a little, but our qPCR would not detect such a small difference.

3)-c: Another method of assessing the effect of perforin on cells is based on permeabilization by trypan blue or staining with propidium iodide. However, it is not generally applicable to EVs. As we mentioned in our response to question 6 from reviewer#1, we need new technologies to examine EV destruction by perforin in more detail.

4) Materials and methods: section about western blotting lacks some basic information, such as the blot type, transfer system and ECL kit used.

**Response:** We thank the reviewer for this comment. We have revised the text as follows: “Samples were prepared in 4× Laemmli Sample Buffer (Bio-Rad Laboratories, California, USA) containing 2-mercaptoethanol (Bio-Rad Laboratories).” (see “Immunoblot analysis” in the “Materials and methods" section; p.8, lines 128–130).

We have also added the following text: “Equal amounts of protein were separated on SDS-PAGE gels using a Mini-PROTEAN® Tetra Vertical Electrophoresis Cell (#1658004JA, Bio-Rad Laboratories) and a PowerPac™ HC power supply (#1645052, Bio-Rad Laboratories). Next, the proteins were transferred to a PVDF membrane (#1620174, Bio-Rad Laboratories) by wet transfer (Mini Trans-Blot® Module, #1703935JA; Bio-Rad Laboratories). Membranes were blocked using Blocking One (NACALAI TESQUE, INC., Kyoto, Japan).” (p.8, lines 130–136).

“The chemiluminescence reagent used to visualize β-actin and Alix wasas Western Lightning Plus-ECL (PerkinElmer, Massachusetts, USA). ImmunoStar LD (FUJIFILM Wako Pure Chemical Corporation, Osaka, Japan) was used to visualize other proteins. ” (p.9, lines 146–149).

5) Fig.3 legend text tells that tryptophan is increased, but the main text and the figure bar graph show that it is decreased. This should be corrected.

**Response:** This error has been corrected. The text has been revised as follows: “(B) The amounts of tryptophan (p = 6.4E-5) and kynurenine (p = 0.010), components of tryptophan catabolism. Kynurenine increased significantly in the presence of IFN-γ.” (Legend to Fig. 3; p.18, lines 309–311).

6) Overall, the discussion is well written and addresses several different factors in the tumor microenvironment. The discussion also refers to an earlier publication (ref. 40) showing enrichment of adenosine in urine and platelet EVs. The same publication also contained a preliminary study of different normalization methods and metabolite changes of urinary EVs in prostate cancer. There, adenosine was found to be decreased in cancer (pre-prostatectomy) samples compared to healthy controls or post-prostatectomy samples. In the light of the results about perforin-mediated lysis of EVs and release of adenosine in the tumor microenvironment, there is a possibility that this phenomenon is reflected also in the urinary EVs in prostate cancer thus supporting the conclusions by Tadokoro et al.

**Response:** We wish to express our deep appreciation to the reviewer for his insightful comment. In accordance with the reviewer's comment, we have revised the text as follows: “Puhka et al. reported that adenosine levels in prostate cancer (pre-prostatectomy) samples were lower than those in healthy controls or post-prostatectomy samples [41]. Considering their results alongside ours, there is a possibility that this phenomenon is reflected also in urinary EVs in prostate cancer patients.” (See "Discussion" section; p.30, lines 513–516).

**References**

1.	Kosaka N, Iguchi H, Hagiwara K, Yoshioka Y, Takeshita F, Ochiya T. Neutral Sphingomyelinase 2 (nSMase2)-dependent Exosomal Transfer of Angiogenic MicroRNAs Regulate Cancer Cell Metastasis. J Biol Chem. 2013;288: 10849–10859. doi:10.1074/jbc.M112.446831

2.	Tominaga N, Kosaka N, Ono M, Katsuda T, Yoshioka Y, Tamura K, et al. Brain metastatic cancer cells release microRNA-181c-containing extracellular vesicles capable of destructing blood–brain barrier. Nat Commun. 2015;6: 6716. doi:10.1038/ncomms7716

3.	Kosaka N, Iguchi H, Yoshioka Y, Takeshita F, Matsuki Y, Ochiya T. Secretory Mechanisms and Intercellular Transfer of MicroRNAs in Living Cells. J Biol Chem. 2010;285: 17442–17452. doi:10.1074/jbc.M110.107821

4.	Villarroya-Beltri C, Baixauli F, Mittelbrunn M, Fernández-Delgado I, Torralba D, Moreno-Gonzalo O, et al. ISGylation controls exosome secretion by promoting lysosomal degradation of MVB proteins. Nat Commun. 2016;7: 13588. doi:10.1038/ncomms13588

5.	Tecalco-Cruz AC, Cruz-Ramos E. Protein ISGylation and free ISG15 levels are increased by interferon gamma in breast cancer cells. Biochem Biophys Res Commun. 2018;499: 973–978. doi:10.1016/j.bbrc.2018.04.030

6.	Liu Y, Zhang T, Zhou Y, Li J, Liang X, Zhou N, et al. Visualization of perforin/gasdermin/complement-formed pores in real cell membranes using atomic force microscopy. Cell Mol Immunol. 2018 [cited 14 May 2019]. doi:10.1038/s41423-018-0165-1

7.	Shi L, Mai S, Israels S, Browne K, Trapani JA, Greenberg AH. Granzyme B (GraB) autonomously crosses the cell membrane and perforin initiates apoptosis and GraB nuclear localization. J Exp Med. 1997;185: 855–66. doi:10.1084/jem.185.5.855

8.	Chen J-H, Xiang J-Y, Ding G-P, Cao L-P. Cholangiocarcinoma-derived exosomes inhibit the antitumor activity of cytokine-induced killer cells by down-regulating the secretion of tumor necrosis factor-α and perforin. J Zhejiang Univ Sci B. 2016;17: 537–44. doi:10.1631/jzus.B1500266

9.	Boison D, Scheurer L, Zumsteg V, Rulicke T, Litynski P, Fowler B, et al. Neonatal hepatic steatosis by disruption of the adenosine kinase gene. Proc Natl Acad Sci. 2002;99: 6985–6990. doi:10.1073/pnas.092642899

10.	Borowiec A, Lechward K, Tkacz-Stachowska K, Składanowski AC. Adenosine as a metabolic regulator of tissue function: production of adenosine by cytoplasmic 5’-nucleotidases. Acta Biochim Pol. 2006;53: 269–78. Available: http://www.ncbi.nlm.nih.gov/pubmed/16770441

11.	Zhong XZ, Zou Y, Sun X, Dong G, Cao Q, Pandey A, et al. Inhibition of Transient Receptor Potential Channel Mucolipin-1 (TRPML1) by Lysosomal Adenosine Involved in Severe Combined Immunodeficiency Diseases. J Biol Chem. 2017;292: 3445–3455. doi:10.1074/jbc.M116.743963
